# Intense pulsed light-based treatment for the improvement of symptoms in glaucoma patients treated with hypotensive eye drops

**DOI:** 10.1186/s40662-022-00284-4

**Published:** 2022-04-01

**Authors:** Jose Maria Martinez-de-la-Casa, Carlos Oribio-Quinto, Almudena Milans-del-Bosch, Pilar Perez-Garcia, Laura Morales-Fernandez, Javier Garcia-Bella, Jose Manuel Benitez-del-Castillo, Julian Garcia-Feijoo, David P. Piñero

**Affiliations:** 1 Ophthalmology Unit, Department of Ophthalmology and ORL, Faculty of Medicine, Hospital Clinico San-Carlos, Universidad Complutense de Madrid, Instituto de Investigación Sanitaria del Hospital Clinico San-Carlos (IdISSC), 28040 Madrid, Spain; 2 Department of Immunology, Ophthalmology and ORL, School of Medicine, Instituto de Investigaciones Oftalmologicas Ramon Castroviejo, Universidad Complutense de Madrid, Madrid, Spain; 3 Department of Optics, Pharmacology and Anatomy, University of Alicante, Alicante, Spain

**Keywords:** Glaucoma, Intense pulsed light, Dry eye, Meibomian glands

## Abstract

**Background:**

Ocular surface disease in glaucoma patients is a significant ocular co-morbidity that can affect 40% to 59% of these patients worldwide. The current study was aimed at evaluating the potential clinical benefit of an intense pulsed light (IPL)-based treatment in glaucomatous patients with ocular surface disease due to prolonged hypotensive eyedrop treatments. To our knowledge, this is the first series analyzing the therapeutic effect of this treatment option in this type of patients.

**Methods:**

This non-comparative prospective case series study enrolled a total of 30 glaucoma patients ranging in age from 57 to 94 years old and treated with hypotensive eyedrops for years with dry eye symptomatology. All patients received four sessions of IPL treatment using the Optima IPL system (Lumenis, Yokneam, Israel) adjusted to the official optimized Lumenis setting. Changes in symptomatology, corneal staining, conjunctival hyperemia, non-invasive break-up time (NIBUT), tear osmolarity, tear meniscus height (TMH), meiboscore and meibomian gland expressibility was analyzed after treatment.

**Results:**

Statistically significant reductions were observed after IPL treatment in the symptomatology scores measured with different questionnaires [ocular surface disease index (OSDI), standard patient evaluation of eye dryness (SPEED) and symptom assessment questionnaire in dry eye (SANDE)] as well as with the visual analogue scale (*P* < 0.001). Mean change in OSDI was − 15.0 ± 11.3. A significant reduction was found after treatment in the corneal staining score (*P* < 0.001). A significant reduction was found in tear film meniscus height (*P* = 0.012), as well as in tear film osmolarity (*P* = 0.001). A significant reduction was also found in meibomian gland expressibility (*P* = 0.003), changing the percentage of grade 3 eyes from 44.4% before IPL to 17.2% after treatment.

**Conclusions:**

IPL therapy combined with meibomian gland expression (MGX) seems to be an effective option to improve symptomatology in glaucomatous patients with ocular surface disease due to prolonged hypotensive eyedrop treatments, with an additional improvement in clinical signs, such as tear osmolarity and corneal staining.

## Background

Chronic glaucoma patients are routinely subjected to topical hypotensive treatments for years [1]. These treatments usually contain preservatives that may have a negative impact on the ocular surface, including alterations of the meibomian gland morphology and function [2, 3], increased subbasal nerve tortuosity and dendritic cell density [4], and affectation of the viability and functions of the conjunctival Goblet cells [5]. These pharmacologically-induced alterations of the ocular surface lead to a significant reduction of the tear film lipid layer [6], and consequently can lead to evaporative dry eye syndrome. This condition is associated to disturbing symptomatology [7] that can affect the patient's quality of life significantly [8]. For this reason, it must be treated with some medical therapeutic approaches proposed, such as the topical use of a preservative-free ophthalmic solution containing hyaluronic acid 0.4% and taurine 0.5% [9], vitamin A palmitate eye gel 0.1% [10], carbomer eye gel 0.2% [10], cannabinomimetic palmitoylethanolamide [11], or oral supplementation with antioxidants and essential fatty acids [12].

In previous years, intense pulsed light (IPL) therapy has demonstrated its effectiveness for the treatment of the dry eye associated to meibomian gland dysfunction (MGD); a recent report from the American Academy of Ophthalmology stated that the existing scientific literature on IPL treatment of MGD confirms the efficacy of this therapeutic option, with improvements in the signs and symptoms associated to this condition [13]. Specifically, the efficacy of IPL for the treatment of dry eye associated to MGD without [14–21] and with the combined meibomian gland expression (MGX) [22–27] has been investigated and confirmed in a great variety of studies. This efficacy is the result of the various therapeutic effects of this irradiation of filtered polychromatic broad-bandwidth wavelengths with varying pulse duration, including the facilitation of the expressibility and release of the meibum inside, the improvement of the function of meibomian glands, the reduction of proinflammatory mediators contributing to dry eye, or the improvement of the cellular functions including fibroblasts regeneration, collagen synthesis, and motility in immunoregulatory cells [19, 28]. These changes induced by IPL facilitates an improvement of ocular surface anomalies or even their resolution, suggesting that the use of this therapeutic approach might be potentially useful in glaucoma patients with an altered ocular surface due to topical hypotensive treatments for years. The aim of this study was to investigate the effect of an IPL-based treatment combined with MGX in glaucomatous patients with pharmacologically-induced moderate to severe ocular surface disease due to prolonged hypotensive eyedrop treatments.

## Methods

### Patients

This non-comparative prospective case series study enrolled a total of 30 glaucoma patients treated with hypotensive eyedrops for at least two years with dry eye symptomatology. The study was conducted at the Department of Ophthalmology of the Hospital Clínico San Carlos in Madrid (Spain) following the tenets of the Declaration of Helsinki. All patients were informed about the nature of the study and provided written informed consent before being included in the trial. The study was approved by the Clinical Investigation Ethics Committee of the San Carlos Clinic Hospital in Madrid (20/588-E).

The following inclusion criteria were defined for this study: chronic glaucoma being treated with at least one hypotensive eye drops for at least two years without changes during the six months before patient's enrolment, patient's ability to read, understand and sign an informed consent form, patient's ability and willingness to comply with the program and the requirements of the treatment, age > 18 years, and two of the following conditions revealing the presence of ocular surface disease:

- Ocular surface disease index (OSDI) questionnaire score ≥ 23 (moderate to severe symptomatology)
- TBUT (tear film break-up time) ≤ 7 s in the studied eye
- MGD score ≤ 12 (evidence of meibomian gland obstruction along the lower eyelid) in the studied eye [29]
- At least 5 non-atrophied meibomian glands along the lower lid of the studied eye
- Tear film osmolarity ≥ 310 mOsm/l in both eyes
- Standard patient evaluation of eye dryness (SPEED) questionnaire score ≥ 10.

Patients with moderate or severe dry eye disease and MGD associated with chronic use of topical hypotensive drugs were included. In all cases, no other causative ophthalmological or systemic pathologies were present, such as Sjögren's syndrome, inflammatory diseases (pemphigus and pemphigoid), infectious (Staphylococci, Demodex folliculorum) or other diseases of the ocular surface.

The exclusion criteria of the study included: skin type V or VI according to Fitzpatrick classification, contact lens wear in the month prior to the baseline evaluation of the current study, ocular or eyelid surgery within 6 months prior to the baseline evaluation of the study and other uncontrolled eye disorders affecting the ocular surface (i.e., active allergies), precancerous lesions, skin cancer or pigmented lesions in the planned treatment area, uncontrolled infections or uncontrolled immunosuppressive diseases, subjects with ocular infections within 6 months prior to baseline examination of the study, previous history of herpes simplex virus 1 and 2, systemic lupus erythematosus, and porphyria, use of photosensitive medications and/or herbs that may cause sensitivity to 560–1200 nm light exposure within 3 months of baseline examination, such as isotretinoin, tetracycline, doxycycline, or St. John's wort, previous facial treatment with IPL within 12 months of evaluation, and not wanting or being able to refrain from the use of medications known to cause dryness (e.g., isotretinoin, antihistamines) for the duration of the study.

### Clinical protocol

Once the informed consent was signed and a patient’s auto-evaluation of symptomatology using the OSDI, symptom assessment questionnaire in dry eye (SANDE) and SPEED questionnaires as well as a visual analogue scale (VAS; scale: 0 to 10), and a complete baseline examination was performed in all patients by a masked examiner including the following tests or clinical evaluations: three consecutive measures of NIBUT (Keratograph 5 M, Oculus Optikgerate, Wetzlar, Germany), slit lamp biomicroscopy (corneal and conjunctival staining evaluated with the Oxford scoring system, 0 to 15; limbar and bulbar conjunctival hyperemia grading), manifest refraction, corrected distance visual acuity measurement using an ETDRS chart, Goldmann tonometry, and infrared meibography (Keratograph 5 M, Oculus Optikgerate, Wetzlar, Germany), grading the meibomian gland dropout degree for each eyelid as meiboscore [30]: grade 0 (no loss of meibomian glands), grade 1 (loss of < 33% of the whole glands area), grade 2 (loss area between 33% and 67%), and grade 3 (loss of > 67% of the whole area). The meiboscore of each eye was calculated as the sum of the scores from both upper and lower eyelids. Likewise, the ability of five meibomian glands in the central area of the lower eyelid was tested for meibum secretion after applying firm digital pressure. The results were scored from 0 to 3 depending on the number of expressible glands found among the 5 central glands, where 0 = all glands expressible; 1 = 3–4 glands expressible; 2 = 1–2 glands expressible; and 3 = no glands expressible.

Immediately after the baseline measurement, the studied eye was determined as the eye with the most severe TBUT (lowest value). If both eyes of the subject had identical TBUT values, the studied eye was determined randomly. Each subject underwent 4 treatment sessions at 2-week intervals and a follow-up session at 4 weeks after the final treatment session.

The first treatment session took place within one week of selection. In addition to this first treatment session, there were three additional treatment sessions at 2-week intervals. The subject could advance a treatment session up to 3 days or delay it up to 7 days. Each treatment session included the following procedures in a sequential order:

1. Slit lamp biomicroscopy (observation of eyelid margins, conjunctiva, and eyelashes).
2. IPL active treatment.
3. MGX of the upper and lower eyelids in both eyes.
4. Slit lamp biomicroscopic evaluation after treatment.
5. Assessment of skin reaction between 5 and 10 min after IPL

There was a single follow-up visit 4 weeks after the last treatment session. The subject could advance the follow-up visit session by up to 3 days or delay it by up to 7 days. At the beginning of the follow-up visit, the subject self-assessed their symptoms using two tools, the OSDI questionnaire and the ocular dryness score using a VAS. A masked examiner conducted the same clinical evaluations that were performed in the baseline examination.

### Treatment sessions

All patients received four sessions of IPL treatment using the Optima IPL system (Lumenis, Yokneam, Israel) adjusted to the official optimized Lumenis setting (590 nm cutoff filter, triple pulses of 6 ms with an interval of 50 ms, and total fluence ranging 11 to 14 J/cm^2^). Before initiating the treatment, each patient underwent a Fitzpatrick skin typing test [31] to determine the intensity of the pulsed light that would be administered.

At each treatment session, the patient was placed in a special chair to perform the treatment, allowing to maintain a comfortable position. The skin was cleaned with micellar water and both eyes of the patient were closed and sealed with special adhesive patches (IPL-aid disposable eye shields, Honeywell Safety Products, Smithfield, USA). A layer of conductive gel for IPL was placed afterwards following the path of the skin on the lower eyelids from temple to temple, including the nose. A total of 5 impacts were then made in each region (right and left), with a total of 10 impacts in each application without overlapping them. After this, another series of a total of 10 impacts was applied again. Finally, MGX was performed using sterile forceps in the slit lamp after instilling anesthetic drops (0.4% oxybruprocaine hydrochloride).

### Statistical analysis

The SPSS software package (SPSS Version 20.0; IBM Corporation, Armonk, NY, USA) was used to analyze the data obtained in this study. Normality of data was first evaluated using the Kolmogorov-Smirnov test. The paired Student t-test and Wilcoxon tests were used for analyzing the statistical significance of the differences between pre-treatment and post-treatment visits when the data samples were normally and not normally distributed, respectively. The Pearson or Spearman correlation coefficients were calculated to assess the degree of association between the change obtained in different variables and the magnitude of baseline parameters depending on whether the data samples were or not normally distributed, respectively. A *P* value < 0.05 was considered as representative of statistical significance.

## Results

### Demographics

A total of 30 eyes of 30 patients with ages ranging from 57 to 94 years old [mean ± standard deviation (SD): 74.6 ± 9.0 years; median: 75.0 years] were analyzed in our study. The sample included 22 females (73.3%) and 8 males (26.7%). Likewise, a total of 16 and 14 right and left eyes were included, respectively. A total of 1, 2 and 3 different types of hypotensive drops were prescribed in 21 (70.0%), 7 (23.3%) and 2 eyes (6.7%), respectively. The Fitzpatrick test confirmed the following distribution of the types of skin: 1 patient (3.3%) skin type I, 5 patients (16.7%) type II, 23 patients (76.7%) type III and 1 patient (3.3%) type IV. Table 1 shows the pre- and post-treatment data for all the variables evaluated in the current sample.

**Table 1:** Summary of the pre- and post-treatment clinical data

Parameters	Pre-treatment Mean (SD) Median (Range)	Post-treatment Mean (SD) Median (Range)	*P* value
OSDI	37.6 (16.6) 34.7 (5.0 to 78.0)	22.6 (14.6) 18.3 (0.0 to 50.0)	< 0.001
SPEED score	13.0 (4.9) 12.5 (6.0 to 25.0)	7.3 (4.8) 6.0 (0.0 to 22.0)	< 0.001
SANDE frequency score	63.8 (22.5) 72.5 (10.0 to 100.0)	37.8 (24.2) 34.2 (0.0 to 95.0)	< 0.001
SANDE severity score	58.6 (22.9) 59.2 (10.0 to 94.1)	35.3 (21.7) 30.0 (0.0 to 85.0)	< 0.001
Symptomatology VAS	5.9 (2.1) 5.5 (1.0 to 10.0)	4.1 (1.7) 4.0 (1.0 to 7.0)	< 0.001
Bulbar hyperemia grading	1.6 (0.5) 1.5 (0.9 to 3.4)	1.4 (0.5) 1.3 (0.5 to 2.3)	0.101
Nasal limbar hyperemia grading	1.2 (0.6) 1.1 (0.4 to 2.7)	1.1 (0.5) 0.9 (0.3 to 2.2)	0.316
Temporal limbar hyperemia grading	1.3 (0.4) 1.3 (0.4 to 1.9)	1.1 (0.6) 1.0 (0.4 to 2.9)	0.123
Oxford staining score	8.9 (2.7) 9.0 (5.0 to 15.0)	6.9 (2.3) 7.0 (3.0 to 12.0)	< 0.001
Tear film osmolarity (mOsm/l)	330.1 (21.1) 328.5 (279.0 to 378.0)	313.8 (21.6) 317.0 (249.0 to 353.0)	0.001
Meiboscore	2.4 (0.6) 2.0 (1.0 to 4.0)	2.2 (0.7) 2.0 (1.0 to 4.0)	0.185
Meibomian gland expressibility	2.4 (0.5) 2.0 (2.0 to 3.0)	2.1 (0.4) 2.0 (1.0 to 3.0)	0.003
Tear film meniscus (μm)	288.5 (169.4) 242.5 (100.0 to 781.0)	229.9 (112.0) 208.5 (129.0 to 710.0)	0.012
First break NIBUT	6.0 (3.7) 5.3 (2.1 to 17.4)	7.4 (3.8) 6.7 (1.9 to 15.1)	0.091
Average NIBUT	13.2 (4.6) 14.2 (4.2 to 21.6)	14.0 (5.1) 15.3 (2.0 to 21.5)	0.348

*OSDI *= ocular surface disease index; *VAS* = visual analogue scale; *NIBUT* = non-invasive break-up time

Regarding the type of hypotensive drug used, the following distribution was found in the sample evaluated: 18 eyes (60.0%) treated with prostaglandin monotherapy, 3 eyes (10.0%) with fixed combination of beta blocker and carbonic anhydrase inhibitor, 2 eyes (6.7%) with fixed combination of beta blocker and prostaglandin, 3 eyes (10.0%) with fixed combination of beta blocker and carbonic anhydrase inhibitor and prostaglandin, 1 eye (3.3%) with fixed combination of beta blocker and carbonic anhydrase inhibitor and brimonidine, 1 eye (3.3%) with beta blocker, 1 eye (3.3%) with carbonic anhydrase inhibitor, and 1 eye (3.3%) with carbonic anhydrase inhibitor and prostaglandin. Mean number of drops per day was 1.53 (SD: 0.9, range 1–4) and mean number of drops with preservatives per day of 0.80 (SD: 1.0, range 0–4).

### Changes in symptomatology

**Fig. 1:**
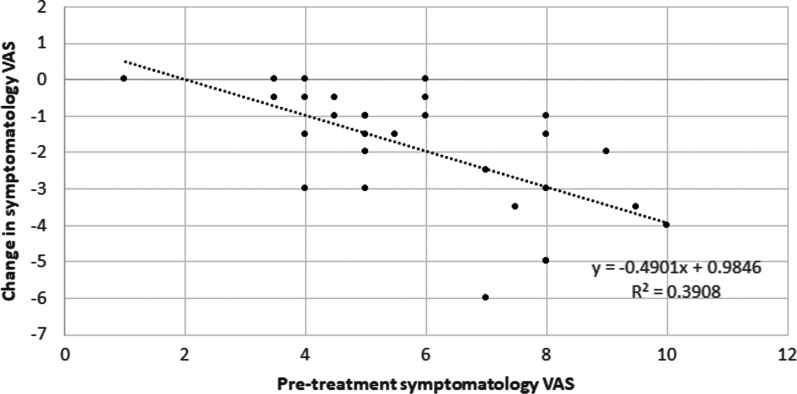
Scatter plot showing the relationship between the change in the score obtained with the symptomatology visual analogue scale (VAS) after intense pulsed light treatment and the pre-treatment symptomatology VAS score. The best fit line to the data obtained by means of the least-squares fit is shown

Statistically significant reductions were observed after IPL treatment in the symptomatology scores measured with different questionnaires as well as with the VAS (*P* < 0.001). Mean change was − 15.0 (SD: 11.3; median − 12.5; range, − 44.5 to 2.5), − 5.6 (SD: 4.2; median -5.5; range, − 17.0 to 0.0), − 26.0 (SD: 19.9; median − 20.9; range, − 73.0 to 0.0), and − 23.4 (SD: 23.1; median − 18.0; range, − 72.0 to 20.0) for OSDI, SPEED, SANDE frequency and SANDE severity scores, respectively. Mean change obtained with the VAS was − 1.6 (SD: 2.3; median − 1.5; range, − 6.0 to 7.0). Inverse significant correlations were found between the change in each symptomatology score and the baseline pre-treatment value (OSDI, r =  − 0.50; SPEED, r =  − 0.44; SANDE frequency, r =  − 0.35; SANDE severity, r =  − 0.55; VAS, r =  − 0.63; all *P* < 0.001, Fig. 1).

### Changes in slit lamp biomicroscopic signs

No significant changes were observed in either levels of bulbar and limbar hyperemia after IPL treatment (*P* ≥ 0.101). In contrast, a significant reduction was found after IPL in the corneal staining score (*P* < 0.001). A statistically significant inverse correlation was found between the changes in the corneal staining score and the pre-treatment level of corneal staining (r =  − 0.50, *P* < 0.001).

### Tear film changes

**Fig. 2:**
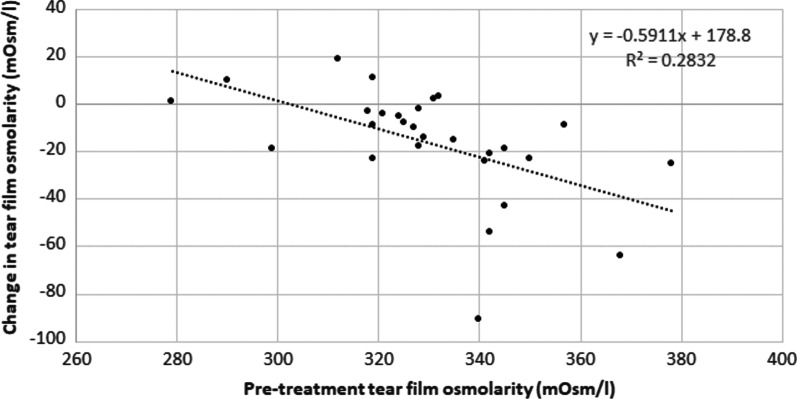
Scatter plot showing the relationship between the change in tear film osmolarity after IPL treatment and the pre-treatment osmolarity value. The best fit line to the data obtained by means of the least-squares fit is shown

A significant reduction was found in tear film meniscus height (*P* = 0.012), whereas no significant changes were found in first break and average NIBUT values (*P* ≥ 0.091). Furthermore, a significant reduction was observed after treatment in tear film osmolarity (*P* = 0.001), with an inverse correlation between the change induced with treatment in this parameter and the baseline value (r =  − 0.53, *P* < 0.001, Fig. 2).

### Changes in morphology and functionality of meibomian glands

**Fig. 3:**
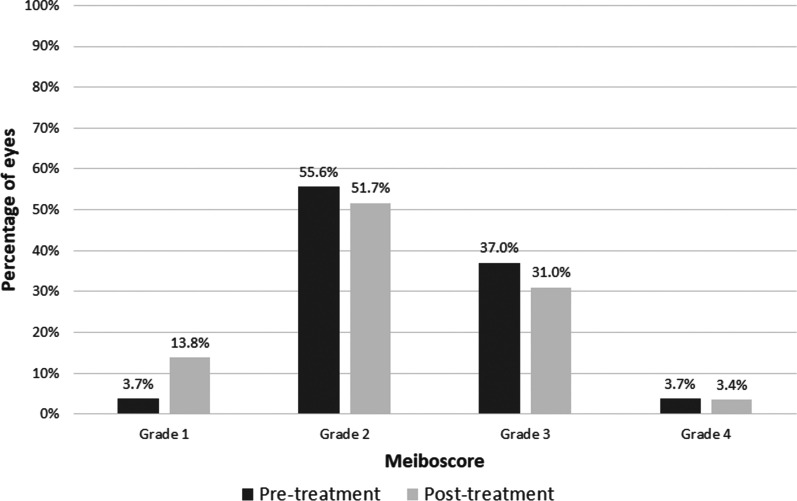
Distribution of the level of meibomian gland loss by meiboscore before and after intense pulsed light treatment

**Fig. 4:**
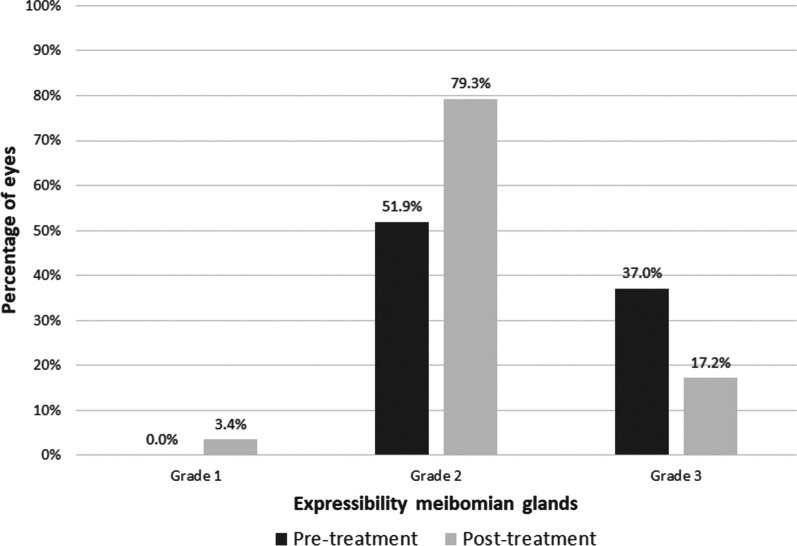
Distribution of meibomian gland expressibility before and after intense pulsed light treatment

No statistically significant reduction after IPL treatment was found in meiboscore (*P* = 0.185). The percentage of grade 3 and 4 eyes according to the meiboscore changed from 40.7% before IPL to 34.4% after treatment (Fig. 3). Concerning meibomian gland expressibility, a significant reduction with treatment was found (*P* = 0.003), with a change of the percentage of grade 3 eyes from 44.4% before IPL to 17.2% after treatment (Fig. 4).

## Discussion

Ocular surface disease in glaucoma patients is a significant, yet often underdiagnosed, ocular co-morbidity that can affect 40% to 59% of these patients worldwide [32]. The current study was aimed at evaluating the potential clinical benefit of an IPL-based treatment combined with MGX in glaucomatous patients with ocular surface disease due to prolonged hypotensive eyedrop treatments. To our knowledge, this is the first series analyzing the therapeutic effect of this treatment option in this type of patients. One of the most consistent outcomes obtained was the significant improvement in symptomatology evaluated with different types of questionnaires, including a VAS. This agrees with the results of previous investigations reporting an improvement of symptomatology evaluated by means of the OSDI questionnaire in dry eyes with MGD [15, 17–21, 33, 34]. This improvement in symptoms has also been observed in cases of MGD associated with rosacea and recently in allergic keratoconjutivitis [35]. To our knowledge, there are no studies on the efficacy of IPL in other types of ocular surface diseases. The absence of other types of ocular surface diseases in the patients included in the present study corroborates the results of IPL in patients with MGD.

The results from the current series are consistent with those reported by other authors evaluating other options of treatments in glaucoma patients with ocular surface disease [36–39]. Boso and colleagues [36] found a significant improvement in OSDI score using as treatment option the combination of eyelid hygiene, fluorometholone acetate 0.1%, preservative-free lubricants, free-acid supplementation and oral tetracycline derivate. Jin and Jin [38] also reported significant improvement in OSDI score after treatment of the ocular surface in medicated glaucoma patients with diquafosol. In our series, the change in symptomatology after IPL with the questionnaires used showed an inverse significant correlation with the level of baseline symptomatology score, with more potential of improvement in those eyes with severe dry eye-related disturbances. This confirms the therapeutic benefit of this treatment option especially in those eyes needing an especially relevant improvement of the symptoms.

The change in symptomatology was associated with several significant changes in ocular signs, such as the reduction of corneal staining and tear film osmolarity. These changes were also inversely correlated with the baseline values of these parameters, confirming that those eyes showing more corneal staining and with higher levels of osmolarity experienced a more significant reduction, as observed with the symptomatology scores. Our results in terms of tear film osmolarity contrasts with those reported by Vigo et al. [16] who did not find significant differences in tear osmolarity, but after three IPL sessions. However, as in our series, Vergés et al. [19] found in a sample of MGD-associated dry eyes treated with IPL that tear osmolarity experienced a significant reduction between baseline and final visit (316 ± 18 mOsm/l vs. 301 ± 12 mOsm/l, *P* < 0.007). Possibly, the reduction in tear film osmolarity achieved with IPL was due to, among other factors, a significant modification of the lipidic secretions from meibomian glands, which led to a more stable lipid layer, better control of the concentration of electrolytes in the aqueous phase of the tear film, and a reduction of the incidence of corneal staining. It should be considered that the regulation of osmolarity has a great impact on the dry eye inflammatory cycle, leading to significant reduction of inflammatory markers in tears (especially IL-17A and IL-6), as demonstrated in previous reports [16, 39]. This regulation of osmolarity should have had a significant impact also on bulbar and limbar hyperemia, but the change did not reach statistical significance, probably due to the intrinsic effect of some antiglaucomatous drugs in ocular hyperemia.

As an improvement in meibomian secretions are related to a more stable tear film, significant increases in NIBUT measures were expected. Indeed, several studies have reported significant reductions in the measurement of NIBUT after IPL, but it should be considered that the follow-up and method used to measure the NIBUT differ significantly among studies [14–19, 32, 33, 40]. Craig et al. [14] reported a significant increase in NIBUT from baseline to the end of IPL sessions in a sample of dry eye subjects participating in a contralateral study, but the tear evaporation rate did not differ significantly between treated and control eyes at any visit. In the sample evaluated, no significant changes were found in first break and average NIBUT values. Ocak and colleagues [17] found that eyes with mild and moderate meibomian gland dropout atrophy did not have an immediate effect on OSDI scores and NIBUT, starting the improvement at 1 month. It should be noted that more than half of the sample had a grade II meibomian gland loss in our series. The limitation of the follow-up may be a factor explaining the non-significant increase in NIBUT, but other factors should be considered as the limitation regarding the consistency of NIBUT measures. Hong et al. [41] confirmed that the coefficient of variation and intraclass correlation coefficient values of NIBUT measured with the instrument used in the current series were 12.8% and 0.93, respectively, for intraobserver repeatability and 15.4% and 0.88, respectively for interobserver repeatability. In any case, IPL may additionally reduce the symptoms and some findings of ocular surface disease through its anti-inflammatory action [42]. Gao et al. [42] demonstrated that IPL can downregulate the levels of IL-17A and IL-1β in tears of patients with evaporative dry eye better than a treatment of anti-inflammatory drops. This action combined with some level of improvement of meibomian secretions could explain the global effect of the therapy, but future studies are needed to prove this hypothesis.

Concerning meniscus height, it experienced a significant reduction after IPL treatment that can be related with reduced reflex tearing observed as the patient's discomfort or the reduction of the meniscus increase associated to the instillation of treatment eye drops [43]. The findings after IPL in terms of tear meniscus height are contradictory, with authors reporting no significant changes [44] and others reporting a significant increase [45]. More studies are still needed to better understand the real impact of IPL combined with MGX in the configuration of the tear meniscus.

Finally, as expected, a significant improvement was found in the level of meibomian gland expressibility after IPL (lower grading, more expressibility), with a change in the percentage of grade 3 eyes from 44.4% before IPL to 17.2% after treatment. This is consistent with the significant improvements in meibomian quality after IPL reported by other groups [15, 21, 32, 33]. Gupta et al. [15] reported in a multicenter cohort study involving 100 patients with diagnosis of dry eye and MGD treated with IPL that there was a significant decrease in meibum viscosity scoring (mean: − 1.1, range: − 3 to 0) and a significant increase in oil flow score (mean: 0.9, range: − 0.5 to 2.0). Importantly, this improvement has not been reported in glaucoma patients with other modalities of treatment.

The results of this study show a potential new indication for IPL, but more consistent and robust studies, including controlled randomized clinical trials, are needed to corroborate this indication and even new indications [13, 46]. In 2020, a report of the American Academy of Ophthalmology on the use of IPL for MGD [13] concluded that the existing peer-reviewed literature to date have shown improvements with signs and symptoms of MGD.

As any non-comparative case series, the study has several limitations. We cannot conclude definitively on the efficacy of the treatment as a comparative study or clinical trial was not conducted. Likewise, masking was not used to minimize potential bias. The current series can be considered a preliminary study reporting some findings that must be confirmed in future comparative studies. Another limitation is that meibum quality was not evaluated. This parameter should be included in future protocols of clinical trials evaluating specifically the efficacy of this treatment option. Furthermore, information about when the patients complained of dry eye symptoms was unavailable because it was unknown if patients had a prior dry eye disease. Many of them were being treated with drugs for years, and thus it was very difficult to pinpoint if patients already reported symptoms before or after starting treatment. In any case, at the onset of the study, all of them had dry eyes and were using antiglaucoma drops. Finally, due to the limitation of the sample size, a comparison among hypotensive agents to analyze differences in the impact on the ocular surface could not be performed. This should be included in future trials as the potential benefit of IPL may differ depending on the type of antiglaucoma drug promoting the ocular surface disorders.

## Conclusions

IPL therapy combined with MGX seems to be an effective option to improve symptomatology in glaucomatous patients with pharmacologically-induced moderate to severe ocular surface disease due to prolonged hypotensive eyedrop treatments, with an additional improvement in clinical signs, such as tear osmolarity and corneal staining. The potential efficacy of this treatment option must be evaluated further in clinical trials as well as the maintenance in the medium- and long-term outcomes achieved.

## Data Availability

The datasets used and/or analyzed during the current study are available from the corresponding author on reasonable request.
